# Do Other Components of Bedding Dust Affect Sensitisation to House Dust Mites?

**DOI:** 10.5402/2011/426941

**Published:** 2012-01-11

**Authors:** Claire Smith, Thorsten Stanley, Julian Crane, Robert Siebers

**Affiliations:** ^1^Department of Paediatrics, University of Otago, Wellington 6242, New Zealand; ^2^Wellington Asthma Research Group, University of Otago, Wellington 6242, New Zealand; ^3^Department of Medicine, University of Otago, Wellington School of Medicine and Health Sciences, P.O. Box 7343, Wellington South 6242, New Zealand

## Abstract

Bedding dust is a mixture of many components, of which the house dust mite (HDM) allergen, Der p 1, is the most allergenic. There has been little work to investigate the effect of other bedding dust components on HDM sensitisation. The objective of the study was to determine the effect of endotoxin in bedding dust on the allergic response in HDM-sensitised individuals. Twenty-nine house dust mite-sensitised adults were skin prick and allergen patch tested against a sterile solution of their own bedding dust and against a solution containing the same concentration of Der p 1 as the bedding solution for comparison. There was no significant difference in wheal size between the diluted house dust mite solution and the bedding dust in spite of their high levels of endotoxin. Symptomatic subjects had larger, but not statistically significant, responses to commercial house dust mite solution than asymptomatic subjects. Allergen patch test responses were negative in 22/29 of subjects using either bedding dust solutions or comparable diluted house dust mite solutions. An individual's own bedding dust does not appear to contain factors that enhance skin prick test or atopy patch test responses to house dust mites.

## 1. Introduction

Sensitivity to house dust was confirmed in the 1920s by studies showing positive skin prick tests to house dust in many asthmatic individuals [1]. Such studies sparked scientific interest into what specific component of house dust was responsible for the sensitisation but it was not until the 1960s that Voorhorst and colleagues discovered *Dermatophagoides pteronyssinus* to be the major producer of house dust allergen [2, 3].

Since these early studies into house dust mite (HDM) sensitivity, there has been much research into the relationship between HDM sensitivity and atopic diseases which have concluded that initial HDM sensitisation and subsequent exposure are strongly linked to asthma, atopic dermatitis, and allergic rhinitis. In particular, the evidence for asthma is strong enough that a causal relationship is widely accepted [1].

Interestingly, despite HDM exposure being accepted as an exacerbating factor in asthma, studies have failed to find a relationship between levels of HDM allergen in the home and asthma symptom severity. Instead, these studies have found asthma severity to better correlate with levels of bacterial endotoxin in house dust [4–6].

Endotoxins are lipopolysaccharide molecules that are found on the outer cell membrane of Gram-negative bacteria. They are known to be potent inducers of inflammation and are present in house dust [7]. High endotoxin levels in the home have been linked to increased asthma severity [4–6]. A synergistic effect has been noted when sensitised subjects were exposed to both endotoxin and allergens, resulting in significantly augmented inflammatory responses [4, 8, 9]. This suggests that endotoxin exposure may play a role in enhancing the immune response to HDM allergens in asthma.

There have been less studies done on the effect endotoxin has on SPT responses to allergens, although one study showed that the addition of pure endotoxin to Der p 1 resulted in a significant increase in the wheal and flare reaction [10]. Pure endotoxin alone cannot elicit a positive SPT response as the inflammation it induces is not IgE mediated [4]. To our knowledge there have been no published studies on the effect of endotoxin on the APT. Direct cutaneous house dust mite allergen exposure has been associated with exacerbation of atopic eczema, and allergen avoidance has been shown to significantly improve eczema as measured by SCORAD [1]. If endotoxin enhances this effect, the clinical implications could be significant.

The aim of this study was to use skin prick and patch testing to determine if an HDM-sensitized individual is more or less reactive to their own bedding dust containing HDM allergens, endotoxin, and other particles, when compared to a pure HDM allergen solution containing equivalent quantities of HDM allergen, but none of these constituents.

## 2. Methods

### 2.1. Subjects

Twenty-nine HDM positive participants were recruited from Wellington Hospital staff and the general population via posters and emails. A wheal size of ≥5 mm to HDM was designated as the inclusion criteria to ensure that a reaction would be expected to occur to a diluted sample of Der p 1. Both symptomatic and asymptomatic HDM positive individuals were included in the study, with 22/29 having symptoms of asthma, atopic dermatitis, or hay fever. This study was approved by the Central Regional Ethics Committee and all subjects gave written informed consent.

### 2.2. Study Design and Methodology

Dust samples were collected by the volunteers by vacuuming their whole mattress surface for 2 minutes and the dust was collected into 25 *μ*m nylon mesh bags [11]. The whole dust sample was weighed and extracted with Tween-20 containing phosphate-buffered saline (PBS) at a ratio of 0.1 g dust to 1.0 mL of PBS for 30 minutes, centrifuged at 4,000 G, and the supernatants stored at −20°C. The major group 1 allergen (Der p 1) from the HDM, *Dermatophagoides pteronyssinus, *was measured in the supernatants by double monoclonal antibody ELISA (Indoor Biotechnologies, UK) as previously described [11]. Endotoxin was measured in 1 : 500 dilutions of the supernatants by a kinetic chromogenic *Limulus* Amoebocyte Lysate (LAL) method (Bio Whittaker, USA) as previously described [12]. The supernatants of the extracted dust samples were passed through a 5 *μ*m filter and stored at −20°C.

For each individual bedding dust supernatant, a commercial endotoxin-free *Dermatophagoides pteronyssinus *SPT solution was diluted to produce an identical concentration of Der p 1 as the Der p 1 level measured in that supernatant. The undiluted commercial SPT solution had a measured concentration of 42,777 ng Der p 1 per mL.

The study took place between December 2007 and February 2008. Participants were instructed to not take oral antihistamine medications for one week before testing when on long-acting antihistamines, or two days before testing when on short-acting antihistamines. SPT was performed in duplicate during the participant's first visit, using a series of eight different solutions (shown in Table 1) applied on the inner aspect of the left forearm. The order of solutions was kept constant throughout the study. SPT was performed using the standard “prick and lift” technique with 1 mm lancets (Stallergenes Ltd) as previously described [13]. The outlines of all wheals were traced with a fine tip pen, and the imprint was transferred onto paper using Micropore tape. The average diameter of each wheal was estimated by taking the average of the longest diameter and the diameter perpendicular to this. Any pseudopodia present were not included.

At the first visit three APTs were applied on the inner aspect of the right forearm or upper arm using the aqueous solutions on filter papers in 10 mm Finn Chambers. No skin abrasion was used. The solutions used are shown in Table 2. Participants were instructed to avoid getting the patches wet during the time they were applied. Participants were asked to remove the patches after 48 hours. Twenty-four hours later (after removal of patches), any reaction was read. If a reaction was present, photos were taken and a grade was assigned to the reaction using the current ETFAD reading key for APT.

Due to nonnormal data distribution, endotoxin and Der p 1 results were log-transformed and expressed as geometric means with 95% confidence intervals (95% CI). Comparison of wheal sizes was conducted using a paired *t*-test while an unpaired *t*-test was used to compare Der p 1 levels between symptomatic and asymptomatic individuals. Pearson's correlation coefficients were calculated between the bedding endotoxin levels and the difference in wheal sizes in response to bedding dust samples and compared to the commercial Der p 1 allergen solutions. Statistical significance was set at the *P* 0.05 level.

## 3. Results

The mean age of participants was 30.4 years (SD ± 3.5) and 22 were female. All 29 participants were skin prick tested with a set of eight allergen solutions, as shown in Table 1. Participant's wheal sizes for each different solution were taken as the average of the two duplicates. All 29 participants had positive reactions to the full strength dust HDM solution (mean wheal size: 8.09 mm ± 1.18) and all, but one, participant had an average Der p 1 wheal size of ≥5 mm as defined in the inclusion criteria. Der p 1 and endotoxin geometric mean levels in the mattress samples were 22.8 *μ*g/g (95% CI: 14.31–36.22) and 11,017 EU/g (95% CI: 7,604–15,962), respectively.

### 3.1. SPT and APT Results

Table 2 shows that there was no significant difference between mean wheal size for the diluted Der p 1 solution compared to the bedding dust extract for the APT. Correlations between endotoxin levels and wheal sizes induced by the diluted Der p 1 solution and bedding extract were not significant (*r* = 0.16 and 0.04, resp.).

Only seven participants (24%) had any positive reactions to the APT. Of those with reactions, three reacted to the diluted dust mite allergen sample only, whilst one reacted to the dust extract only. All of the four who reacted to only one of the patches had a mild Grade 1 reaction. For the remaining three who reacted to both solutions, two reacted more to the diluted Der p 1 solution, while one reacted more to the dust extract. Combined, there were six positive patch test reactions to the diluted commercial HDM solution (mean grade of 1.7) and four positive reactions to the bed dust solution (mean grade of 1.25), as shown in Table 2. There were no significant differences between the levels of endotoxin or Der p 1 allergen in the patch positive and patch negative individuals' dust (Table 3).

### 3.2. Bedding Dust Der p 1 and Endotoxin

The symptomatic group (*n* = 24) had significantly higher levels of bedding Der p 1 than the asymptomatic group while endotoxin levels were similar between the two groups (Table 4). Mean wheal sizes of Der p 1 were slightly larger on average in the asymptomatic than in the symptomatic group although this did not reach statistical significance (Table 4).

## 4. Discussion

The main findings of our study were that there was no significant difference in SPT responses in Der p 1 sensitised individuals tested with Der p 1 solution compared to a bedding dust sample containing an identical concentration of Der p 1 and high levels of endotoxin. This contrasts to the study by Michel et al. [10] who demonstrated a synergistic enhancement of the SPT response to HDM extract.

However, it is difficult to compare the concentration of endotoxin used in these two studies as Michel et al. used a commercial preparation measured in *μ*g/mL whereas in our study endotoxin activity was expressed as Endotoxin Units. Furthermore, the effects seen by Michel et al. (wheal response of 6.4 mm with endotoxin versus 4.9 mm without) were modest.

It is possible that there are other components in bedding dust that may act as inhibitors of SPT responses in the presence of endotoxin and this may explain our inability to show an effect. The original design of this study was to skin prick test using commercial HDM solution with or without the addition of pure endotoxin. This design would have been more effective in measuring the effects of endotoxin, but we felt that there might be ethical objections to applying pure endotoxin on the skin in volunteers.

There was no correlation found between the levels of Der p 1 in dust and the wheal size to the diluted samples used. This suggests that there may be a threshold effect occurring, where the size of the wheal depends on the allergen concentration only up to a certain point, and where further increases of allergen concentration result in little or no increase in wheal size.

The low levels of positive reactions to the patch tests may have been due in part to the diluted levels of Der p 1 used and the low prevalence of eczema in the sample population (only 37% had ever been diagnosed with eczema). It is unlikely to be due to the methodology as a similar method produced a high frequency of positive tests in a cohort of fifty children with atopic eczema [14]. The finding of some positive reactions to the diluted HDM in those without atopic dermatitis is consistent with other studies, as the APT using HDM is not entirely specific for atopic dermatitis [15]. 

The geometric mean of levels of Der p 1 in bedding dust was less than that seen in previous studies done in the Wellington region [11]. This may be due to chance or to characteristics of the sample population. Samples were also collected in summer which coincides with lower levels of HDM in homes.

The limitations of our study include relatively small numbers of subjects studied and in particular the ability to separately study subjects, who are sensitised but asymptomatic versus those with clinical allergy, especially those with atopic eczema.

In conclusion, an individual's own bedding dust does not appear to contain factors that enhance SPT or APT responses to HDM. Since endotoxin, present in high amounts in individual's bed dust, has been shown to do this in previous studies, other factors present may act as inhibitors of the inflammatory response. Endotoxin in bedding dust does not appear to enhance allergic responses as measured by SPT and APT in HDM-sensitised subjects.

## Figures and Tables

**Table 1:** Skin prick test results.

	Positive control	Negative control	SPT HDM solution	Diluted SPT HDM	Dust extract	SPT cat	SPT grass
Positive reactions	100%	0%	100%	93.1% (27/29)	96.5% (28/29)	65.5% (19/29)	51.7% (15/29)

Wheal diameter mm (95% CI)	5.1 (4.6–5.5)	0	8.1 (6.9–9.3)	5.3 (4.4–6.2)	5.1 (4.3–5.8)	6.7 (5.1–8.3)	4.0 (3.5–4.5)

**Table 2:** Atopy patch test results.

	Diluted SPT HDM	Dust extract
No of positive reactions	6/29 (20.7%)	4/29 (13.8%)

Grade of reactions	Grade 1: 4/6	Grade 1: 3/4
	Grade 2: 1/6	Grade 2: 3/4
	Grade 4: 1/6	

Average grade	1.7	1.25

**Table 3:** APT positive versus APT negative individuals.

	APT positive	APT negative
Number	7/29	22/29

Endotoxin EU/gr	8,556	11,940
Geometric mean (95% CI)	(4,939–14,821)	(7,503–18,714)

Der p 1 *μ*g/gr	27.7	21.4
Geometric mean (95% CI)	(17.1–44.8)	(11.8–38.8)

Eczema	4/7	7/22

**Table 4:** Asymptomatic versus symptomatic individuals.

	Asymptomatic	Symptomatic
Number*	5/28	22/28

Wheal size mm	7.6	10.5**
	(5.7–8.6)	(5.3–15.7)

Endotoxin EU/gr	10,430	15,200**
Geometric mean (95% CI)	(6,620–16,432)	(8,128–28,415)

Der p 1 *μ*g/gr	10.0	27.0***
Geometric mean (95% CI)	(5.4–18.6)	(15.3–46.7)

*Symptom information from one individual unavailable.

**P > 0.05.

***P = 0.04.
